# *TP53* Abnormalities and MMR Preservation in 5 Cases of Proliferating Trichilemmal Tumours

**DOI:** 10.3390/dermatopathology8020021

**Published:** 2021-05-25

**Authors:** Raquel Martín-Sanz, José María Sayagués, Pilar García-Cano, Mikel Azcue-Mayorga, María del Carmen Parra-Pérez, María Ángeles Pacios-Pacios, Enric Piqué-Durán, Jorge Feito

**Affiliations:** 1 Ophthalmology Department, Complejo Asistencial Universitario de Salamanca, 37007 Salamanca, Spain; rmartinsan@saludcastillayleon.es; 2 Pathology Department, Complejo Asistencial Universitario de Salamanca, 37007 Salamanca, Spain; ppmari@usal.es (J.M.S.); mcparra@saludcastillayleon.es (M.d.C.P.-P.); mapacios@saludcastillayleon.es (M.Á.P.-P.); 3 Plastic Surgery Department, Complejo Asistencial Universitario de Salamanca, 37007 Salamanca, Spain; pgarciac@saludcastillayleon.es; 4 Pathology Department, Hospital José Molina Orosa, 35500 Arrecife, Spain; mazcmay@gobiernodecanarias.org; 5 Dermatology Department, Hospital José Molina Orosa, 35500 Arrecife, Spain; 6 Human Anatomy and Histology Department, Universidad de Salamanca, 37007 Salamanca, Spain

**Keywords:** trichilemmal cyst, proliferating trichilemmal tumor, MMR, *TP53*, p53

## Abstract

Proliferating trichilemmal tumours (PTT) are defined by a benign squamous cell proliferation inside a trichilemmal cystic (TC) cavity. A possible explanation of this proliferative phenomenon within the cyst may be molecular alterations in genes associated to cell proliferation, which can be induced by ultraviolet radiation. Among other genes, alterations on *TP53* and DNA mismatch repair proteins (MMR) may be involved in the cellular proliferation observed in PTT. Based on this assumption, but also taking into account the close relationship between the sebaceous ducts and the external root sheath where TC develop, a MMR, a p53 expression assessment and a *TP53* study were performed in a series of 5 PTT cases, including a giant one. We failed to demonstrate a MMR disorder on studied PTT, but we agree with previous results suggesting increased p53 expression in these tumours, particularly in proliferative areas. *TP53* alteration was confirmed with FISH technique, demonstrating *TP53* deletion in most cells.

## 1. Introduction

Trichilemmal cyst (TC) is a benign cyst derived from the external root sheath of the catagen hair follicle isthmus by a budding-off mechanism [1]. TCs are generally located on the scalp and have a slight predilection for females [2,3].

Proliferating trichiilemmal tumour (PTT) was described in 1966, accounting for 2% of the TC, and has been initially considered a variant of these trichilemmal cysts [4,5]. This tumour has been described under different denominations, but PTT is the currently accepted term [4,6]. PTTs are defined histologically by an endophytic proliferation, sometimes with a lobulated pattern, giving a multiloculated or solid aspect [4]. They have a homogenous keratinization, lacking a defined granular layer, similar to TCs. Calcification is a common finding [4,7]. As the TCs, the PTTs are usually located in the scalp, have slight predilection for females and have a variable size, ranging from very small lesions to giant tumours [4]. Although they were proposed to develop from TC [4], recent studies propose a de novo genesis [8].

Aggressive, malignant or even metastasizing variants of PTT have been described [7,9,10]. Malignant degeneration can be present over a preexisting PTT [11,12], involving an infiltrating pattern with variable cytological atypia [9,13]. Malignant cases may have differential diagnosis issues regarding either keratinizing squamous cell carcinoma or trichilemmal carcinoma [9,14,15]. The cytokeratin immunohistochemical (IHQ) pattern is not helpful in distinguishing these entities [16]. However, Ki67 has demonstrated an intermediate proliferation rate in PTT, in a range between squamous carcinoma and TC, suggesting PTT is not a trivial cyst. It is, instead, a low-grade neoplasm with a potentially aggressive clinical course [6,17,18,19]. Ki67, altogether with CD34 and p53, may aid in differentiating PTT from squamous cell carcinoma [14].

Regarding the molecular mechanisms responsible for developing these cystic lesions, genetic susceptibility was described for simple TC in some cases: hereditary cases may develop with an autosomal dominant type of transmission, involving incomplete penetrance [1,20,21]. TC development has been recently related with phospholipase C delta 1 (PLCD1), probably via an inherited PLCD1 high-risk allele and somatic spontaneous mutation on the other allele [20,21]. Very little mutation analysis has been performed on PTTs: there were described genetic anomalies, mainly aneuploidy, within some PTT [17]. Mutations were noted on locus 17p13, where *TP53* gene resides [18,19]. Some reports had related these genetic alterations with malignant change [22,23], associated occasionally with *TP53* deletion [12]. The precise mechanism responsible of the proliferative ability of PTT has been not yet clarified, but the accumulation of mutations on tumour suppressor genes seems a possible option [20].

Mismatch repair (MMR) is one of the cellular mechanisms involved in the repair of DNA injuries, caused by insertions or deletions produced during DNA replication by DNA polymerases [24]. A mutation on these genes is constitutively present in the cells of Lynch syndrome patients, and also in its variant associated with cutaneous sebaceous neoplasms, Muir-Torre syndrome [25,26]. This mutation is also present in other carcinomas including up to 15% of sporadic colorectal adenocarcinomas [24,27]. MMR can be routinely diagnosed by IHQ targeted against the key proteins MSH2, MSH6, MLH1 and PMS2, or directly by testing microsatellite instability (MSI): both techniques have high sensitivity in detecting both Lynch syndrome and the sporadic forms of MSI [28]. Our lab employs MMR IHQ as long as morphology is preserved on the slide.

Regarding skin tumours, the significant relationship between sebaceous tumours and Muir-Torre syndrome makes neccesary to look for abnormalities regarding MMR expression when diagnosing these tumours [29,30], although MMR abnormalities per se do not permit the distinction between Muir-Torre associated and sporadic sebaceous tumours [31]. Keratoacanthomas are also associated with Muir-Torre syndrome [26]; these tumours are more frequent and usually do not demonstrate MMR alterations when studied in the general population [32]. However, in patients with Muir-Torre syndrome, a MMR alteration can be found in keratoacanthomas, and also in other premalingnant lesions such as actinic keratosis (AK) or Bowen’s disease [33]. Other cutaneous tumours where the role of MMR proteins has been studied are melanocytic tumours [34], squamous neoplasms [35] and even simple TC [36], showing reduced expression in melanocytic lesions and no evidence of mutation in squamous carcinoma or trichilemmal cysts. MMR positive neoplasms are a promising target of immunotherapy in the future [37].

Ultraviolet (UV) ratiation, in particular UVB, is responsible of cutaneous neoplasms development, via direct DNA damage [38]. *TP53* has been particularly linked with UV damage, and there are ‘signature’ *TP53* mutations in skin cells and tumours that are highly associated with UV damage [38,39]. MMR, the other studied repair mechanism has a less evident relationship with UV, but there is indirect evidence supporting its role in UV repairment [38]. However, although its mutation is likely to contribute to squamous cell carcinoma (SCC) progression, MMR deficiency is unlikely to play a significant role in SCC carcinogenesis [40]. 

The usual location of PTT is the scalp [11], although PTT is an odd lesion that has been described in unsuspected areas, like glabrous skin (i.e., skin devoid of terminal hair follicles) [41] or other regions distant from the head [42]. In the usual scalp location these tumours can reach giant dimensions [42,43,44,45,46,47]. The preference for a cephalic location suggests a possible role of UV in the development of trichilemmal lesions. 

Finally, although PTT is not a sebaceous lesion, they have a common embriologic origin and hair follicle anatomy indicates that the isthmus region where TC and PTT develop is closely related to the sebaceous gland [48]. On one hand, the sebaceous gland may be the target to obtain hair follicle stem cells from the isthmus and bulge [49]. On the other hand, bulge cells can be mobilised to maintain sebaceous glands [48], crossing the isthmus for this purpose [2]. Besides, there are reports of PTT with divergent differentiations, including sebaceous [50] or apocrine [50,51]. Furthermore, there are also examples of association between sebaceous carcinoma and PTT, with no clinical evidence of Muir-Torre or Lynch syndrome [52]. Based on these assumptions, we wanted to search for alterations in the IHQ expression of p53 and MMR proteins, also looking for deletion of *TP53* in these tumours via a FISH assay, in an attempt to explain their proliferating nature.

## 2. Materials and Methods

A series of five PTT cases diagnosed in the last 6 years were included in the study, with cases from José Molina Orosa Hospital (n = 4) and a giant case from Complejo Asistencial Universitario de Salamanca (n = 1), (Table 1). 

**Figure 1:**
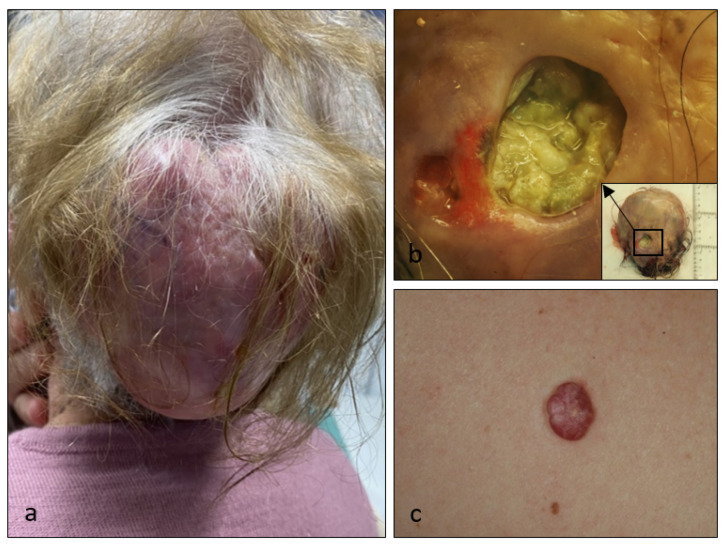
Clinical and macroscopic images of proliferating trichilemmal tumours. There is depicted Case 1 before the surgical procedure (**a**) and in the pathology laboratory, before fixation, where a dilated pore was prominent (**b**). There is also depicted Case 2 in the back (**c**).

Most patients had non ulcerated multilobed masses that were resected with clinical suspect of trichilemmal cyst (Figure 1a). On occasion a dilated pore was present, guiding the diagnosis (Figure 1b). The exception is Case 2, where fibroma or Pinkus fibroepitelioma were the clinical diagnoses (Figure 1c). None of the studied cases had cutaneous malignant events and no posterior recurrence was found (with at least 1 year follow-up).

Samples were routinely embedded in paraffin for light microscopy. Paraffin blocks were cut into 3 µm sections and stained with haematoxilyn-eosin. IHQ was performed in a Bond III platform (Leica, Wetzlar, Germany) using prediluted antibodies from Leica Biosystems, directed against p53 (clone DO-7, Leica Biosystems, prediluted), MSH2 (code PA0989, clone 79H11), MSH6 (code PA0990, clone EP49), MLH1 (code PA0988, clone ES05) and PMS2 (code PA0991, clone EP51). Images were taken with an Olympus BX53 microscope connected to an Olympus UC90 camera (Olympus, Tokyo, Japan), with no ulterior image proccessing. After conventional histological studies, a copy number alterations *TP53* study was performed using fluorescence in situ hybridization (FISH), with a probe specific for the *TP53* gene located in chromosome 17p13 and another directed to the centromere of chromosome 17 (17p11.1) (Vysis, Downers Grove, IL, USA). The number of hybridization spots was evaluated using a DMRB fluorescence microscope (Leitz, Wetzlar, Germany) equipped with a 100× oil objective, which was used for counting the number of hybridization spots/cell in at least 200 cells/sample. 

## 3. Results

**Figure 2:**
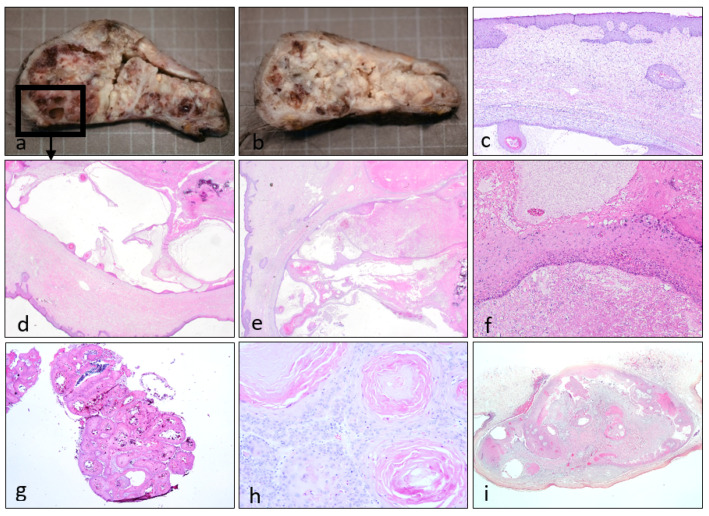
Correlation between macroscopic aspect after fixation and histological images. On the giant lesion (Case 1, **a**–**f**) was appreciable the multiloculated aspect, alternating whitish keratotic content and more solid lesions (**a**,**b**). Histologically the lesion presented a hyperplastic epidermis and an underlying cystic lesion with epithelial endophytic projections and anastomosing epithelial strands (**b**–**e**); image “d” corresponds to the macroscopic lesion marked in image “a”. On some areas trichilemmal epithelium (left side) presented focally a prominent granular layer (right side), with compact keratinization and parakeratosis (**f**). Histologic findings were variable, with marked calcification and minor keratin content (Case 5, **g**). In the solid areas maturation was preserved, with reactive changes and minor inflammation (Case 3, **h**). Tumoral silhouette generally revealed a sharply defined cystic lesion, with proliferation towards the interior of the cyst (Case 2, **i**).

The studied tumours showed a well-defined cystic architecture, with several proliferative changes and a trichilemmal squamous lining (Figure 2). Detailed examination of the epithelium demonstrated no pleomorphism, although occasional reactive features may be noted in the more solid regions (Figure 2h). 

**Figure 3:**
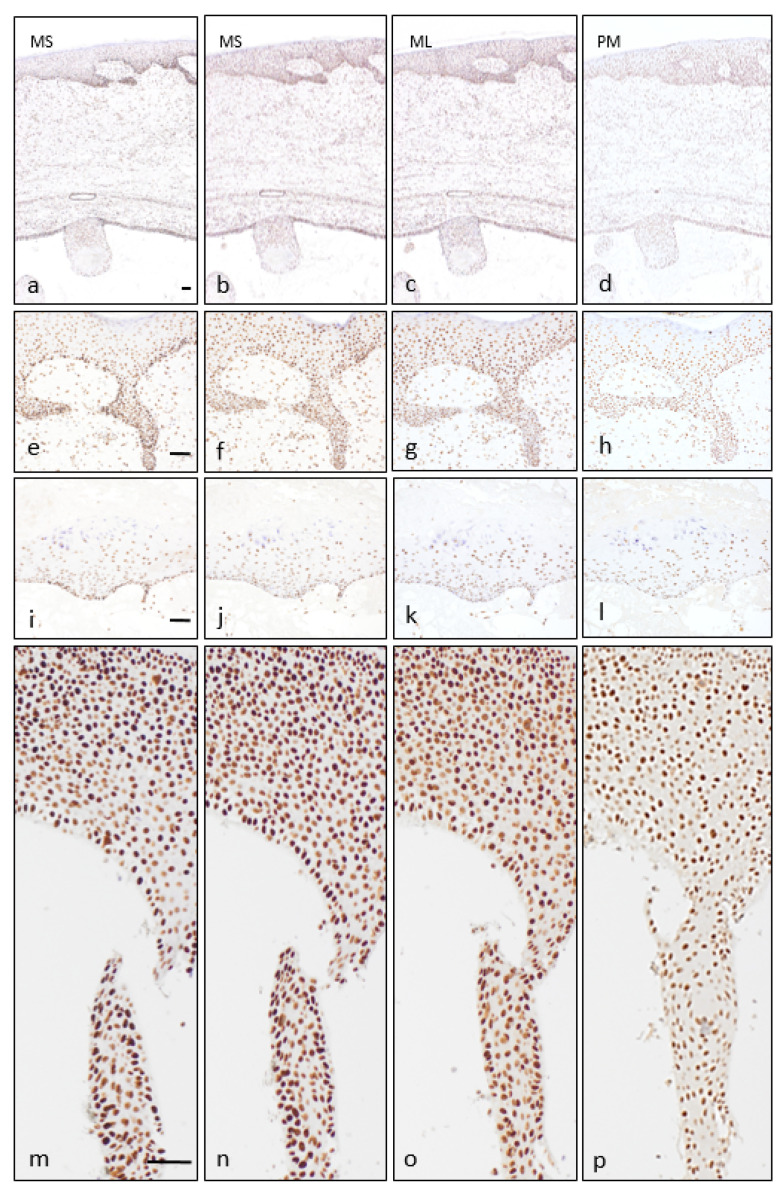
Mismatch repair protein expression. There are depicted immunohistochemical results for MSH6 (**a**,**e**,**i**,**m**), MSH2 (**b**,**f**,**j**,**n**), MLH1 (**c**,**g**,**k**,**o**) and PMS2 (**c**,**h**,**l**,**p**) in different regions of Case 1 to illustrate their variability. Tumour can be identified in the inferior part of the image, with slightly hyperplastic overlying skin (**a–d**). On a detailed view, overlying skin displayed widespread nuclear expression with positive control in dermal inflammatory cells (**e**–**h**). On the tumour there were present areas with reduced nuclear expression in the thinnest epithelial lining with focal hypergranulosis (**i**–**l**), contrasting with the increased expression in proliferative areas (**m**–**p**). Scale bars (**a**–**d**, **e**–**l**, **m**–**p**): 50 µm.

The four MMR proteins (MSH2, MSH6, MLH1, and PMS2) were expressed in all the studied tumours, in both tumour epithelium and normal covering skin (Figure 3a–d). Most cells demonstrated nuclear expression of the four MMR proteins in the epidermis and also in the hair follicles (Figure 3e–h); this expression was especially evident on epidermal basal and squamous layers, declining in the granular layer. The proliferative cystic epithelium had regions with sparser expression than epidermis (Figure 3i–l) while other more cellular areas demonstrated widespread positivity (Figure 3m–p). Results are semiquantitatively summarized in Table 2. 

**Figure 4:**
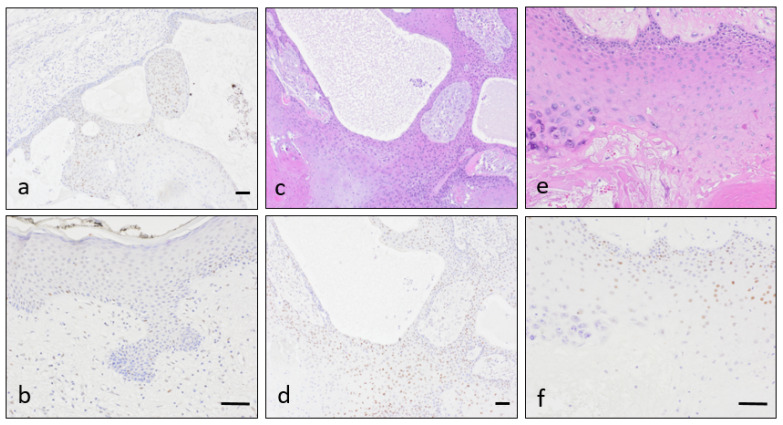
p53 expression on Case 1, illustrating its variability. There was weak nuclear p53 expression in the more cellular areas of the proliferating tumor (**a**), while in the overlying epidermis there was very scarce nuclear expression (**b**). There were regional differences regarding p53 expression pattern, being more obvious in the more solid and cellular regions (**c**,**d**) and less apparent when the epithelial lining was thinner or hypergranulotic (**e**,**f**). Scale bars (**a**,**c**,**d**; **b**,**e**,**f**): 50 µm.

IHQ for p53 showed nuclear expression on many tumoral cells (Figure 4a), while overlying skin had no expression or was expressed by fewer cells (Figure 4b). It was apparent in a greater amount in the more cellular areas (Figure 4c–d). On the other hand, p53 exhibited decreased immunostaining in areas of parakeratosis, contrasting with the increased expression where trichilemmal keratinisation occurred (Figure 4e–f). Even when observing the less cellular areas, proliferative tumour had a higher degree of p53 expression than overlying skin.

**Figure 5:**
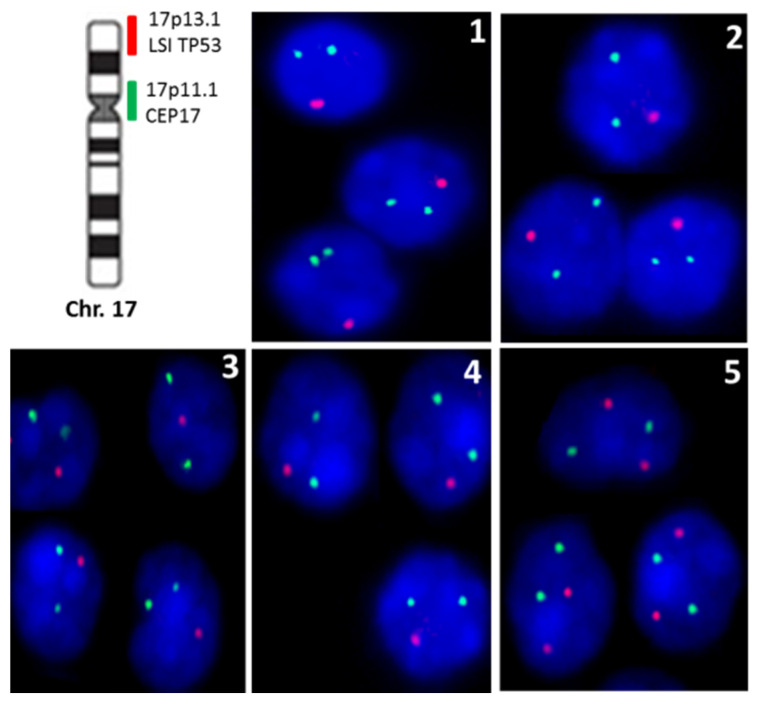
Representative pictures of cell nuclei from 5 PTT patients after hybridization with two probes for chromosome 17 (17p11.1 and 17p13; green and red signals, respectively). Four tumors showed loss of TP53 gene (Cases 1–4). Case 5 displayed a normal diploid number of hybridization signals for the both probes analyzed.

17p13 deletions were displayed by 4 of the 5 PTT patients included in the current study in >50% of the studied nuclei per case (Figure 5). The remaining patient (Case 5) showed two copies of chromosome 17p13 per nuclei.

## 4. Discussion

UVB radiation is the main carcinogenic agent in the epidermis, being able to cause various mutations regarding both *TP53* gene and also in MMR, originating MSI [38,39]. MMR and *TP53* disarrangements have already been described in lung carcinogenesis [53] and their mutation have summatory effects reducing chemotherapy effect on colonic adenocarcinoma [54]. In normal skin the most important protein concerning UV-induced tumorigenesis is p53 [38], and the relevance of the MMR system remains unclear [39].

The studied tumours present areas of both increased and decreased MMR expression compared with normal epidermis of the scalp. Increased expression is morphologically related with the most proliferative regions of the tumor and decreased expression is observed usually in thin epithelial strands. We consider the result is not enough relevant, as long as intense nuclear expression of the MMR proteins can be observed in most epithelia and no homogeneous pattern of expression could be identified. In any case, the high expression in the most cellular (and probably proliferative) regions compared with the not-so-cellular areas may be the expected result, considering the previously reported overexpression in other squamous premalignant lesions [55,56]. 

MMR system is also likely to be a component in UVB-induced DNA damage repairment, and over-expression has been observed on occasion in SCC [57], although others failed to demonstrate a significant relationship [40]. MSH2, one of the principal components of the MMR system, is part of the *TP53* repair mechanisms [55]. MSH2 is also elevated in premalignant cutaneous neoplasms, but its increased expression in SCC is less evident [55]. Furthermore, similar findings are present in other squamous lesions like cervical intraepithelial neoplasia (CIN) and carcinoma: MMR are elevated in preinvasive lesions, but they are not increased in malignant neoplasms [56]. This finding seems biologically rational, involving a higher rate of DNA repairment in premalignant lesions.

Moreover, MMR alterations are typical of sebaceous neoplasms. MMR-intact sebaceous neoplasms tend to be located in the head region (where sebaceous tumours are more prevalent), while MMR-mutated ones are, by contrast, frequent outside the head [58]. This supports the idea that MMR mutation loses importance when talking about neoplasms of the head region, and it is a striking thinking, as long as the head region is indeed very exposed to UV at the same time the hypothesis of an UVB related disorder on MMR proteins has been already noted [55]. A recent paper by North et al., [59] explains this apparent contradiction, caused by three mutually exclusive mutations leading to sebaceous carcinoma: the first is a MMR-mutated profile and the second is a MMR-intact one, with UV damage-associated mutations, already described in the literature [58]. The third possible mutation was described as pauci-mutational and is present in some facial tumours [59]. It has been proposed to develop from sun-shielded epithelial cells [59], which are likely to correspond to Meibomian glands. This third group of mutations can explain the low incidence of MMR mutations on sebaceous tumours in the head when compared with extracephalic ones.

In our hands, p53 expression taken alone can be interpreted either as increased or within the wild-type spectrum [60]. This result might be considered in line with an increased DNA repair status in the context of a premalignant tumour. The difference between superficial epidermis and PTT is remarkable (Figure 4a,d): p53 expression is discrete in some PTTs, but skin covering the tumor shows nearly absent expression. However, the immunohistochemical result may be considered positive and suggestive of mutation in some tumour spots [61]. FISH assay demonstrates deletion of *TP53* in most cells in 80% of our small series, so the overexpression is not only reparative, but indicative of a DNA damage leading to *TP53* deletion.

*TP53* is one of the most widespread genetic alterations of human oncogenesis, and its mutation usually confers higher oncogenic potential and poorer prognosis to most neoplasms [62]. UV-related alterations on *TP53* have been extensively described in melanoma and also in non-melanoma cutaneous cancer [38,39]. *TP53* has, indeed, an initial role in cutaneous carcinogenesis in contrast to internal malignancies, where TP53 mutations develop at a later stage [63]. Mutations in the *TP53* gene have been described as an early event in SCC development and, as so, are present in AK as well as in other precursor lesions [64,65]. With the help of other molecules including calcitriol, p53 enhances DNA repair in keratinocytes [63,66]. In normal skin p53 is barely present, but is expressed after UV irradiation, suggesting an initial p53 overexpression to overcome the UV-caused DNA damage [65,66]. In this line, there has already been suggested on occasion a role of p53 alterations in PTT [12,19]. Some authors [19] consider PTT as a carcinoma, due to their similar to SCC p53 profile, while others demonstrated *TP53* mutation in a malignant PTT [12]. This fact could be in accordance with the premalignant nature of PTT, but in contrast to AK where mutations occur in *TP53* [67], we demonstrate a *TP53* deletion in several benign appearing PTT. In this context, it would be interesting to sequence TP53 searching for UVB-signature mutations in PTT, clearly linking to sunlight exposure [63].

Although as general rule TP53 deletion implies impaired p53 expression [68], the relationship between increased p53 expression and TP53 deletion has been already evaluated in esophageal squamous carcinoma, where a correlation between both was present but not complete [69]. This correlation is specially known in haematological neoplasms [70] like multiple myeloma, where hemizygous *TP53* mutation is associated with increased p53 nuclear expression due to mutations in the remaining allele [71,72]. In this disease, *TP53* alterations can occur in three subsets: monoallelic deletion, monoallelic mutations and biallelic inactivation, where monoallelic deletion is the only currently associated with a high risk profile [73].

A detailed genetic study was performed once on a case of malignant PTT, which demonstrated mutated TP53 in both malignant and non malignant tumours. In that case the malignant tumour also showed allelic loss on chromosome arm 17 [12]. Our case series has an example of a non-deleted case, but the other four cases demonstrate deletion, two with weak overexpression of p53 and another two with absent p53. The presence of constitutive deletion in the majority of PTT in our series supports the hypothesis of de novo generation of PTT [8].

## 5. Conclusions

In summary, we have found no relevant alterations in the expression of MMR proteins on the studied cases. In any case, the mismatch repair pathway is not completely understood, and there is an important subset of patients fulfilling criteria for Lynch syndrome that do not have detectable abnormalities on MMR [74]. The *TP53* deletion in 80% of studied cases, with p53 overexpression, is indicative of a serious DNA damage and a potentially aggressive neoplasm. Limitations of the present study are the small number of cases studied and the absence of precise sequentiation.

## Figures and Tables

**Table 1:** Cases included in the study.

Case	Gender	Age	Topography	Size
Case 1	Female	88	Scalp (occipital)	9 cm
Case 2	Female	45	Back	1 cm
Case 3	Female	84	Scalp (frontal)	3 cm
Case 4	Female	63	Scalp (occipital)	3 cm
Case 5	Female	69	Scalp (parietal)	1.5 cm

**Table 2:** Immunohistochemical results of MMR, p53 immunohistochemistry (IHQ) and *TP53* deletion in fluorescence in situ hybridization (FISH).

Case	MSH2	MSH6	MLH1	PMS2	P53 IHQ	*TP53* Loss
Case 1	80%	80%	60%	70%	Weak	63%
Case 2	60%	60%	40%	30%	Negative	65%
Case 3	90%	80%	80%	80%	Negative	52%
Case 4	90%	90%	90%	90%	Weak	80%
Case 5	90%	90%	80%	70%	Negative	0%
